# Real-time monitoring of mitochondrial oxygenation during machine perfusion using resonance Raman spectroscopy predicts organ function

**DOI:** 10.1038/s41598-024-57773-w

**Published:** 2024-03-27

**Authors:** Rohil Jain, Emmanuella O. Ajenu, Manuela Lopera Higuita, Ehab O. A. Hafiz, Alona Muzikansky, Padraic Romfh, Shannon N. Tessier

**Affiliations:** 1 Center for Engineering in Medicine and Surgery, Harvard Medical School and Massachusetts General Hospital, Boston, MA USA; 2 Shriners Children’s Hospital, Boston, MA USA; 3 Department of Electron Microscopy Research, Clinical Laboratory Division, Theodor Bilharz Research Institute, Giza, Egypt; 4 Biostatistics Center, Massachusetts General Hospital, Boston, MA USA; 5 Pendar Technologies, Cambridge, MA USA

**Keywords:** Assay systems, Translational research, Liver

## Abstract

Organ transplantation is a life-saving procedure affecting over 100,000 people on the transplant waitlist. Ischemia reperfusion injury (IRI) is a major challenge in the field as it can cause post-transplantation complications and limit the use of organs from extended criteria donors. Machine perfusion technology has the potential to mitigate IRI; however, it currently fails to achieve its full potential due to a lack of highly sensitive and specific assays to assess organ quality during perfusion. We developed a real-time and non-invasive method of assessing organs during perfusion based on mitochondrial function and injury using resonance Raman spectroscopy. It uses a 441 nm laser and a high-resolution spectrometer to quantify the oxidation state of mitochondrial cytochromes during perfusion. This index of mitochondrial oxidation, or 3RMR, was used to understand differences in mitochondrial recovery of cold ischemic rodent livers during machine perfusion at normothermic temperatures with an acellular versus cellular perfusate. Measurement of the mitochondrial oxidation revealed that there was no difference in 3RMR of fresh livers as a function of normothermic perfusion when comparing acellular versus cellular-based perfusates. However, following 24 h of static cold storage, 3RMR returned to baseline faster with a cellular-based perfusate, yet 3RMR progressively increased during perfusion, indicating injury may develop over time. Thus, this study emphasizes the need for further refinement of a reoxygenation strategy during normothermic machine perfusion that considers cold ischemia durations, gradual recovery/rewarming, and risk of hemolysis.

## Introduction

Currently, over 100,000 individuals anxiously await a lifesaving organ transplant, yet thousands of these will die every year while on the waitlist due to a severe organ shortage. Current clinical standards for organ handling for transplantation include a period of cold ischemia during transport, which is aimed at suppressing metabolism to extend ex vivo graft survival by several hours. Human livers, for instance, can be stored for up to 8 h in static cold storage (SCS) immersed in a preservation solution without additional dissolved oxygen^1,2^. Following cold ischemia, grafts are then exposed to a sudden burst of oxygen supply during implantation, resulting in ischemia–reperfusion injury (IRI). IRI is characterized by a reduction in the ATP levels, accumulation of metabolic waste products^3,4^, and damage to cells mediated by stresses such as ROS that may lead to cell death and organ dysfunction^5,6^. IRI remains a significant challenge in transplantation that is responsible for posttransplant graft dysfunction^7,8^ and is poised to limit the utilization of grafts that are exposed to warm ischemic injury, including donation after circulatory death (DCD)^9,10^.

Several strategies currently exist to mitigate IRI with the goal of reducing (or eliminating) ischemia while minimizing reperfusion injury. Ex situ machine perfusion (MP) is currently the leading strategy that maintains organs in an active state and delivers oxygen and nutrients via a perfusate prior to transplantation. The metabolic activity and oxygen demand during MP can vary considerably depending on the temperature of perfusion. Simultaneously, different perfusate mediums can be altered to supply the desired amounts of oxygen and nutrients. For example, Normothermic Machine Perfusion (NMP), an FDA approved protocol, involves maintaining the organ at a physiological temperature of 37 °C and supplementing the perfusate with an oxygen carrier, such as packed red blood cells (pRBC)^11^, although other synthetic oxygen carriers have also been used experimentally^12,13^. While NMP minimizes cold ischemia time, grafts are continuously maintained in a hyper-oxygenated state^14^. Sub-normothermic machine perfusion (SNMP) is another method that maintains organs at 21 °C with an acellular perfusate carrying only dissolved oxygen that may be sufficient to meet lower metabolic demands. Like NMP, SNMP reduces cold ischemia; however, it uses a more gradual increase in metabolism that may protect injured organs^15^ from a sudden burst in oxygen consumption at 37 °C^16^. Finally, hypothermic oxygenated machine perfusion (HOPE) uses dissolved oxygen in a perfusate pumped at 4–10 °C as a means to prevent early mitochondrial injury upon reperfusion^17,18^, although it represents a departure from physiology.

Despite the protective benefits of these techniques, mechanistic insights into the optimal oxygenation strategy to improve metabolic recovery while minimizing IRI during machine perfusion is lacking in the field. Mitochondria are attractive targets to observe reoxygenation due to their extensive role in metabolism and IRI^19–21^. Measurement of mitochondrial activity can be used to study metabolic recovery after ischemia and reperfusion. Further, injury to mitochondria may also predict cellular damage. Despite the importance of mitochondria, current technologies to measure mitochondrial function during machine perfusion lack sensitivity/specificity, do not capture the dynamic nature of oxygenation as a function of IRI, or rely on destructive tissue sampling^22^. Instead, we developed a metric of mitochondrial function using a real-time, non-destructive measurement of mitochondrial redox state using resonance Raman spectroscopy (RRS)^23^.

The reduced and oxidized states of mitochondrial cytochromes have unique resonance Raman spectral signatures when excited with a 441 nm laser. This spectrum is recorded from the surface of organs during MP by focusing the laser on a 2 mm diameter spot on the left lateral lobe in the region that is in proximity to the portal vein, where the depth of penetration of the laser is estimated to be < 1 mm. This spectrum is simultaneously cross referenced with fully oxidized and reduced mitochondrial cytochrome spectral libraries in real-time, to derive the resonance Raman reduced mitochondrial ratio (3RMR). 3RMR is defined as a ratio of reduced to total mitochondrial complex redox states. In a healthy condition there is a steady transfer of electrons from the mitochondrial complexes to oxygen, which keeps them in a relatively oxidized state, i.e., with low 3RMR. However, such transfer can be disrupted during ischemia and reperfusion and lead to a higher reduced state, i.e., higher 3RMR. In contrast to other methods, the 3RMR level can be used to directly measure local transfer of electrons to oxygen with high sensitivity and specificity. Moreover, it works independently of the presence of hemoglobin, yet provides complementary hemoglobin saturation measurement; and thus, can be used with both RBC-based and acellular perfusates. Finally, it is a non-invasive and real-time assay without cumbersome mitochondrial isolation procedures^24^, making it ideal for longitudinal assessment during machine perfusion.

To leverage this powerful technology to understand IRI and develop an assay of mitochondrial health, we measured 3RMR during normothermic machine perfusion of rodent livers that were exposed to minimal versus extended cold ischemia durations (0-h cold ischemia vs 24-h cold ischemia). We also varied the NMP protocol to include either an acellular perfusate (0hCI Acellular and 24hCI Acellular groups) or packed RBCs (24hCI Acellular and 24hCI pRBC groups) to understand the impact of oxygen delivery methods on ischemia and reperfusion injury. We measured the 3RMR for each group, for a duration of 3 h of NMP with simultaneous measurements of machine perfusion parameters, including oxygen uptake rate (OUR), inferior vena cava (IVC) lactate output, resistance, Alanine Aminotransferase (ALT) level, Aspartate Aminotransferase (AST), among others. Biopsy samples were also obtained at the end of 3 h of perfusion for energetic measurements and histology. We show that while there is no difference in 3RMR of fresh livers comparing acellular- versus cellular-based perfusates, livers stored for 24 h at 4 °C showed a delayed recovery of mitochondrial oxidation state with an acellular perfusate as compared to pRBCs. However, perfusates containing pRBC showed evidence of hemolysis, rising resistance and ALT/AST, and evidence of endothelial damage.

## Results

### RRS allows quantification of mitochondrial redox state with different perfusate compositions

**Figure 1:**
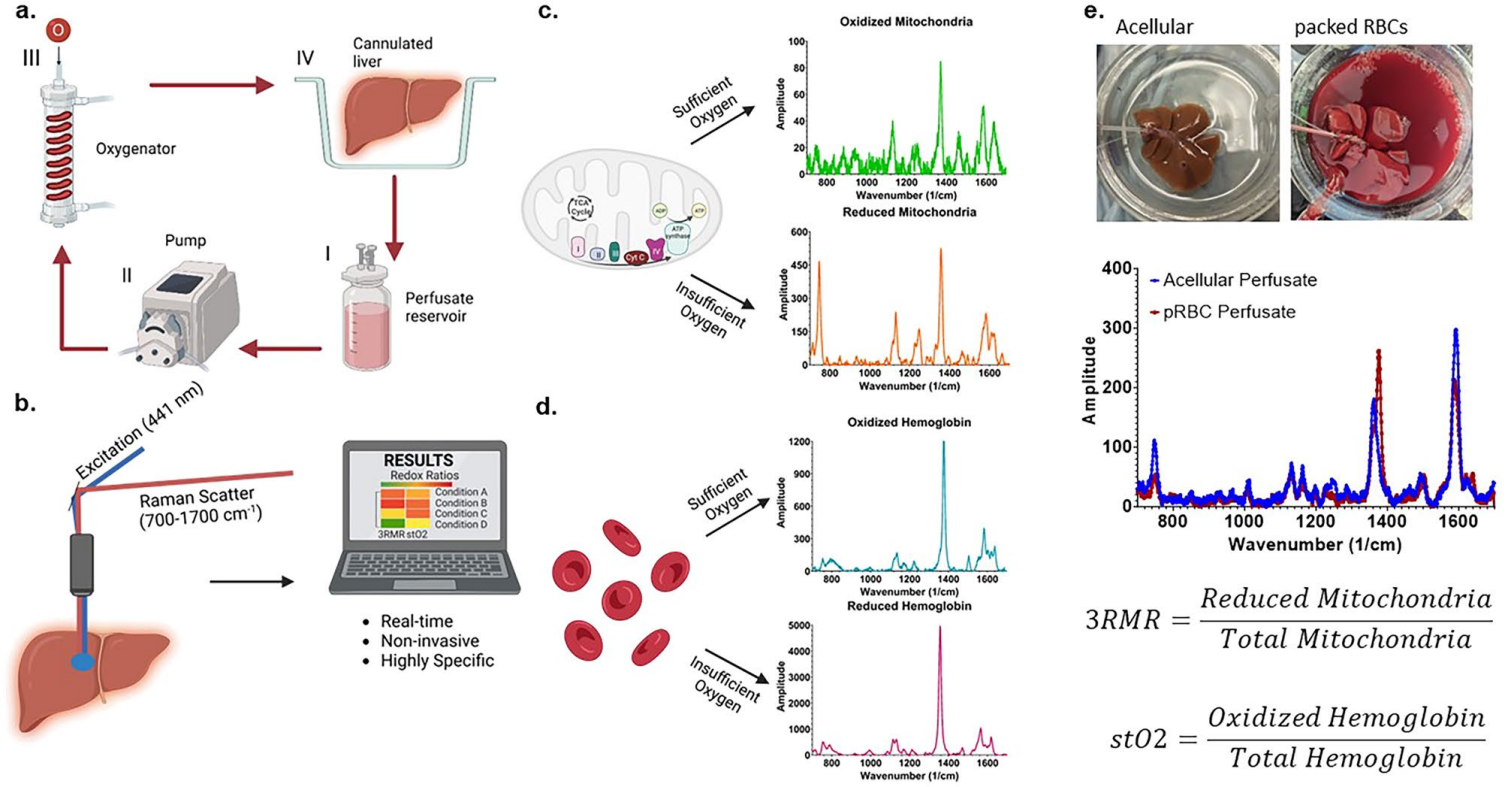
Resonance Raman Spectroscopy predicts functional oxygenation of liver tissue during machine perfusion. (**a**) Schematic of machine perfusion of livers where a perfusate(I) with or without red blood cells as oxygen carriers is circulated by a pump (II) to an oxygenator (III). The oxygenator consists of a gas-permeable tubing that carries the perfusate surrounded by oxygen at a higher ambient partial pressure than atmosphere. This allows the perfusate to be oxygenated before it is supplied to a cannulated liver (IV). (**b**) The principle of our custom approach to measuring mitochondrial redox state in the tissue non-invasively using a resonance Raman spectroscopy device. A 441 nm excitation laser is used to excite molecules with a porphyrin ring (such as mitochondrial complexes, cytochromes, and hemoglobin) that produce a resonance Raman spectrum. (**c**) The spectrum of oxidized and reduced isolated mitochondria. (**d**) The spectrum of reduced and oxidized hemoglobin. (**e**) Two rat livers that are sitting in the perfusion bowl that are supplied with oxygen either with or without RBCs. Directly below the livers are the RRS spectrum from each liver. This spectrum is deconvoluted into its constituent molecular signatures using pre-recorded libraries as shown in c and d. The deconvolution coefficients for the reduced mitochondria is averaged with the sum of reduced and oxidized mitochondria to quantify the redox state of tissue and called the 3RMR value. Similarly, the oxygen saturation of hemoglobin in the tissue is also obtained by taking the ratio of oxidized hemoglobin coefficient with the total coefficients of oxidized and reduced hemoglobin.

RRS allows accurate quantification of the redox state of mitochondria as well as hemoglobin in the perfusate since both mitochondrial complexes and hemoglobin molecules possess a porphyrin ring structure with a strong Soret absorption band at 441 nm wavelength. Excitation near the Soret band results in a resonant enhancement of the vibrational modes that result in Raman spectra. When excited by this laser during machine perfusion (Fig. 1a), the vibrational scattering spectrum from the liver surface is carried via optical fibers to a Charge Coupled Device (CCD) array, and eventually analyzed using custom LabVIEW program (Fig. 1b). This program deconvolves the spectrum into its components by using pre-recorded libraries of fully reduced and oxidized mitochondrial complexes and hemoglobin (Fig. 1c,d) by minimizing error in a statistical regression fit. Depending on the perfusate composition, either only the mitochondrial libraries, or both mitochondrial and hemoglobin libraries are used for calculating 3RMR. Upon deconvolution, weights are assigned to each complex which are then used to calculate the Resonance Raman Reduced Mitochondrial Ratio (3RMR) which is the ratio of the weights of reduced to total mitochondrial 3RMR (Fig. 1e)^23^. Figure 1e also shows representative images of rodent liver perfusions with and without the packed RBCs, and representative spectrum from each type of perfusion. All the RRS measurements were performed on the exposed surface of the left lateral lobe which is in proximity to the portal vein, as determined in our previous study^25^. A more detailed description of the perfusion system and the Raman device is available in the methods section.

### 3RMR reflects oxygenation dynamics and energetic recovery of 0-h cold ischemic and 24-h cold ischemic livers in real time during perfusion

**Figure 2:**
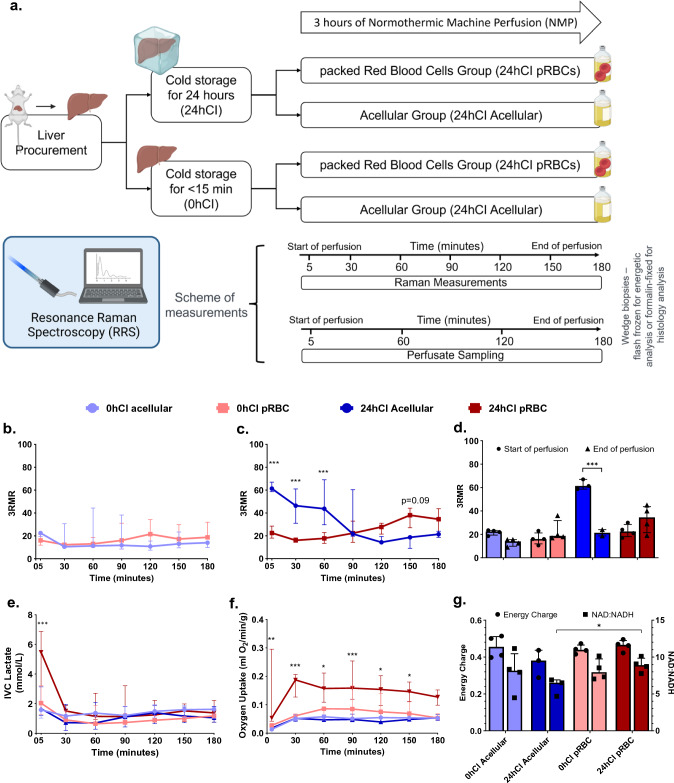
3RMR with lactate, energy charge, and NAD:NADH ratio predict metabolic recovery dynamics during machine perfusion. (**a**) Schematic of the experiments and test groups. Each liver is either stored at 4 °C for 24 h or perfused immediately after recovery from the rat. It may then be machine perfused with either a packed RBC based perfusate or an acellular perfusate for 3 h at 37 °C. During this time the perfusate is sampled every 60 min for blood gas, blood chemistry, and ALT/AST analysis. Raman measurements are also taken from the surface of the livers every 30 min. At the end of the perfusion, wedge biopsies from the liver are either flash frozen for energetic tests or stored in formalin for histology. (**b**) Low 3RMR values throughout the 3 h of perfusion that are statistically indistinguishable between 0hCI acellular (n = 4) and 0hCI pRBC (n = 4) groups. (**c**) Significantly higher 3RMR value immediately after reperfusion of 24hCI acellular group (n = 3) compared to 24hCI pRBC group (n = 4). The 3RMR value decreases continuously during perfusion and becomes statistically indistinguishable around 90 min of perfusion. This trend reverses after 90 min indicating possible dysfunction of mitochondrial electron transport chain, however remaining statistically the same. (**d**) The comparison between 3RMR values at the start of perfusion and at the end of perfusion for each of the tested conditions. The p-values that are marked for each pair show statistically significant lower 3RMR values for acellular perfusate while those with packed RBCs are statistically the same. (**e**) Lactate levels during the 3 h of perfusion for all four conditions. That remain low and indistinguishable for all the tested conditions during perfusion. (**f**) Oxygen uptake rate for all four groups during perfusion. The oxygen uptake for 24hCI pRBC group is the highest which reflects the higher oxygen demand that is satisfied by the higher supply of oxygen bound to RBCs. OUR between the other three groups is comparable with only slightly higher values in the 0hCI pRBC group. (**g**) Energy charge and NAD:NADH values for all the four conditions tested. These were obtained from flash frozen wedge biopsies from the livers at the end of 3 h of perfusion. While not statistically significant, the ratios for machine perfusion of 24-h cold ischemic livers perfused with acellular perfusate were slightly lower than the other conditions, indicating potential slower recovery of oxidative phosphorylation. We plotted median ± IQR for all the variables. Statistical significance levels as follows *p < 0.05, **p < 0.01, ***p < 0.005.

Our study to observe ischemia and reoxygenation during machine perfusion using 3RMR consisted of groups that reflect different degrees of ischemic stress in the form of short or long durations of storage at 4 °C—one group of livers had minimal ischemia (< 15 min), referred to as the 0 h cold ischemic (0 h-CI) livers; and a second group of livers that were stored for 24 h in cold ischemic condition (24 h-CI) livers. These groups were chosen because the former mimics fresh transplantations with minimal ischemia, while latter mimics the longest cold ischemic duration that maintains 100% survival after transplantation based on previous studies at our group^26^. We also further tested two perfusate compositions—one that contained packed RBCs as oxygen carriers (pRBC group) and a second group where no oxygen carriers were used (acellular group), thus making it a total of four experimental groups (0hCI-Acellular, 0hCI-pRBC, 24hCI Acellular, 24hCI pRBC) with 3–4 replicates per group. These perfusate compositions were chosen to understand the effects of different levels of oxygen supply and test the usefulness of the RRS device in predicting mitochondrial oxygenation for each condition. A summary of the storage and perfusion conditions are shown in Fig. 2a.

As shown in Fig. 2b, we observed a low 3RMR value for 0 h-CI livers irrespective of the mode of oxygenation. At the start of perfusion (t = 5 min), the acellular perfusate group showed a median (IQR) of 3RMR as 22. 5 (19.4–23.4), and the pRBC group showed the 3RMR to be 16.0 (11.9–21.3) -which were not statistically different (p > 0.05). The values remained low throughout the 3 h of perfusion, with acellular group 3RMR of 14.0 (9.9–15.5), and pRBC 3RMR of 18.8 (17.3–31.9) (p > 0.05) at the end of perfusion. This suggests sufficient oxygenation with both perfusates, indicating that the dissolved oxygen at a higher partial pressure (500–600 mm Hg) is sufficient for meeting the oxygen demand of these livers. However, this was different from the trend in 24 h-CI group of livers, as shown in Fig. 2c. The 3RMR value was high immediately upon reperfusion in the 24 h-CI acellular group with a median (IQR) of 61.3 (58.7–67.0), which was in contrast with the low 3RMR in the 24hCI-pRBC group of 22.5 (18.0–28.5) (p < 0.05). The high 3RMR may indicate these ischemic livers were insufficiently supplied with enough oxygen by the acellular perfusate compared to the pRBC perfusate. Interestingly, the 3RMR for 24hCI-acellular livers decreases during perfusion to 21.7 (19.7–60.3) at 90 min and remains at a similar level by the end of perfusion at 21.3 (18.7–24.0). The 3RMR for the 24hCI-pRBC group increases marginally at t = 150 min to 38.0 (27.0–44.1) compared to the 24hCI-Acellular group (p = 0.09), however becomes statistically indistinguishable (p > 0.05) by the end of perfusion. This appears consistent with the observation that 24 h cold stored livers can also be fully recovered with sub-normothermic machine perfusion^27^. The 3RMR values at the beginning and the end of perfusions for each group are also summarized in Fig. 2d highlighting significant reduction in 3RMR with the 24 h-CI acellular group (p < 0.05).

The initial period of ischemia and subsequent recovery of 24 h-CI groups also shows interesting trends in other perfusion-based biochemical markers. Figure 2e shows the venous lactate concentration is significantly higher (p < 0.05) at t = 5 min for 24hCI-pRBC group: 5.47 (2.14–6.87) mM, compared to other groups- 24 h-CI acellular: 1.66 (1.21–3.13) mM; 0 h-CI pRBC: 2.04 (1.27–3.22) mM; 0 h-CI acellular: 1.59 (1.03–1.71) mM. It is likely due to lactate production by red blood cells that cannot be delineated from liver-derived lactate. The IVC lactate in the 24 h-CI pRBC group gets cleared by the first 30 min of perfusion (reaching 1.51 (0.93–1.89) mM) and reaches statistically similar levels (p > 0.05) to 24hCI-acellular (0.72 (0.19–1.96) mM) and 0hCI-pRBC (0.91 (0.81–1.50) mM) groups (p > 0.05 for both comparisons). The lactate values remain stable thereafter for the 3 h of perfusion with the following values recorded at t = 180 min: 0 h-CI acellular-1.62 (1.02–1.77) mM; 0 h-CI pRBC-1.18 (1.02–1.23) mM; 24 h-CI acellular-1.05 (0.75–1.81) mM; 24 h-CI pRBC-1.38 (0.75–2.22) mM. Another important metric of oxygenation is shown in Fig. 2f which is called the oxygen uptake rate (OUR). We observe higher oxygen uptake rate in the 24hCI-pRBC group compared to other groups in the initial phase of perfusion, as seen in the following values at t = 5 min of perfusion- 0hCI acellular, 0hCI pRBC, 24hCI acellular, and 24hCI pRBC: 0.013 (0.012–0.015), 0.027 (0.019–0.033), 0.018 (0.013–0.023), and 0.053 (0.042–0.295) ml/min/g respectively (p < 0.05 for 24hCI-pRBC vs all three groups). However, OUR level became lower and statistically similar (p > 0.05) compared to other groups by the end of perfusion (t = 180 min) with OUR: 0hCI acellular, 0hCI pRBC, 24hCI acellular, and 24hCI pRBC: 0.053 (0.047–0.056), 0.053 (0.049–0.069), 0.054 (0.045–0.065), and 0.127 (0.098–0.152) ml/min/g respectively (p > 0.05 for all comparisons). This may indicate an initial higher demand of oxygen due to ischemia in the 24hCI groups, which is quickly met by the supply from the pRBC perfusate. Furthermore, we assessed the correlation of 3RMR with OUR and IVC Lactate, as shown in Supplementary Fig. S3. We observed fair to strong correlations in the 24hCI groups, although it should be noted that lactate is generated by both red blood cells and livers and these perfusions were flow-driven, which may have impacted these correlations.

Finally, Fig. 2g shows energy charge (EC), defined as ratio (ATP + 0.5*ADP)/(ATP + ADP + AMP)^28^ and NAD:NADH ratio for all groups. Energy charge is an indicator of energetic recovery at the end of perfusion, while NAD:NADH ratio is an indicator of utilization of substrates at complex I of the electron transport chain during machine perfusion. EC shows statistically similar levels in all the groups of livers at end of the 3 h of perfusion- 0hCI acellular: 0.46 (0.40–0.51), 0hCI pRBC: 0.44 (0.42–0.47), 24hCI acellular 0.38 (0.29–0.44), and 24hCI pRBC- 0.47 (0.43–0.49) (p > 0.05 for all comparisons). On the other hand, NAD:NADH ratios for each group were 0hCI Acellular: 8.14 (5.34–10.45), 0hCI pRBC: 7.93 (7.10–9.74), 24hCI Acellular: 6.51 (4.68–6.90), and 24hCI pRBC: 8.90 (8.01–9.84), respectively with p > 0.05 for all pairwise comparisons, except 24hCI acellular vs 24hCI pRBC where the difference between groups was statistically significant with p < 0.05. Based on these metabolic markers, it may be hypothesized that 3RMR, ATP, and NAD:NADH ratios confirm energetic recovery for all groups of livers, but perhaps, sub-optimally in the 24hCI acellular perfusion group.

### Increased levels of injury markers indicate suboptimal recovery of livers perfused with pRBCs

**Figure 3:**
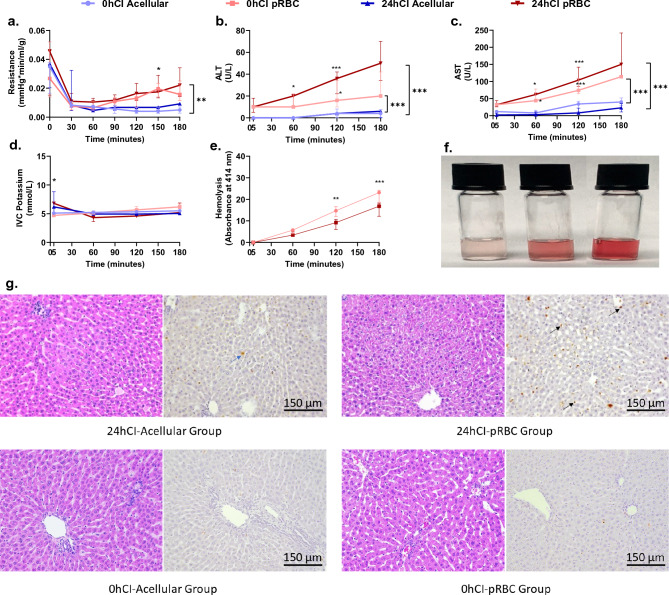
Portal vein pressure, ALT, and AST levels indicate higher injury to livers perfused with packed RBCs compared to acellular perfusate for the 3 h of perfusion. (**a**) Shows the trends in resistance for the tested conditions. It is significantly higher in the pRBC groups than the acellular perfusate groups after 2 h of perfusion. There is no significant difference between the fresh and cold storage groups for each type of perfusate. (**b**,**c**) Trends in ALT and AST levels between the four groups, where pRBC perfusate groups show higher values compared to the acellular perfusate groups. The statistically significant difference becomes more prominent over time for these groups. (**d**) Shows no significant difference in IVC potassium for all four groups during perfusion. (**e**) Shows hemolysis over the duration of perfusion. There is no significant difference at any time during the perfusion between 0 and 24h CI groups. The hemolysis is also evident from the increasing red coloration of the perfusate samples with time as shown in (**f**). Finally, (**g**), shows representative histology to show the patters of injury in 24hCI livers from acellular and pRBC groups stained with H&E and TUNEL stains (Magnification 20x). The blue arrows indicate hepatocyte death while the black arrows show endothelial death as observed via TUNEL staining of apoptotic cells. Each group has a 3–4 replocates. We plotted mean ± standard deviation for AST and hemolysis which followed normal distribution, and median ± IQR for resistance, ALT, and potassium which were observed to not follow a normal distribution. Statistical significance levels *p < 0.05, **p < 0.01, ***p < 0.005.

To quantify injury due to different ischemic durations and perfusate composition, we looked at markers of injury such as portal resistance, alanine aminotransferase (ALT) and aspartate aminotransferase (AST) levels, potassium, hemolysis for pRBC perfusate, and histological markers of injury during and at the end of 3 h of perfusion for all groups. We observed a portal resistance after 3 h that trends towards higher values in the packed RBC groups with the following values- 0hCIAcellular: 0.005 (0.003–0.007, 0hCI pRBC: 0.016 (0.011–0.025), 24hCI Acellular 0.009 (0.001–0.010), 24hCI pRBC: 0.022 (0.014–0.034) mm Hg*min/ml/g; however, only reached statistical significance for the 0hCI acellular vs 24hCI pRBC groups with p < 0.05 (Fig. 3a). ALT and AST levels are considered important markers of liver injury, especially during machine perfusion^29^. At the beginning t = 5 min, these values were as follows for ALT and AST respectively- 0hCI Acellular: 0 (0–0) and 2 (0–7); 0hCIpRBC: 10 (10–10) and 32 (28–36); 24hCI Acellular: 0 (0–0) and 12 (0–16); and 24hCI pRBC: 10 (5–18) and 32 (26–44) U/L, which were statistically indistinguishable with p > 0.05. However, the differences became much more significant by the end of perfusion (p < 0.05) for both ALT and AST, especially in the 24hCI pRBC and 0hCI pRBC groups with the following values at t = 180 min for ALT and AST respectively- 0hCI Acellular: 4 (0.5–7.5) and 23 (11–35); 0hCIpRBC: 20 (20–42) and 114 (114–114); 24hCI Acellular: 6 (0–6) and 40 (30–52); and 24hCI pRBC: 50 (34–70) and 150 (116–242) U/L. The time-varying ALT and AST concentrations for all the groups are shown in Fig. 3b,c, respectively. Finally, hyperkalemia (high potassium) is also a marker of injury to the organ that measures release of potassium into the perfusate due to cell death. The potassium concentration stayed between 4 and 8 mmol/L for all groups during perfusion, without reaching statistical difference (p > 0.05) between any of the tested groups once the perfusion was stabilized (i.e. after t = 30 min) as shown in Fig. 3d.

Hemolysis of RBCs occurs as they are continuously exposed to mechanical stresses due to a peristaltic pump in the machine perfusion setup. The damaged RBC product reduces the oxygen carrying capacity of the perfusate and hemolysis-derived products may be toxic. Thus, we quantified hemolysis by analyzing the absorbance properties of the perfusate at 414 nm wavelength (detailed protocol in methods section). We observed increasing hemolysis with perfusion time in both 0hCI pRBC and 24hCI pRBC groups as seen in Fig. 3e, where the absorbance of 414 nm wavelength of light increases at a steady rate over time and reaching the following values at t = 180 min: 0hCI pRBC: 23.2 (18.6–24.2), and 24hCI pRBC: 16.8 (12.2–18.3) which were statistically different with p < 0.05. The increasing hemolysis can also be observed from the increasing intensity of red coloration of the perfusate, where intact RBCs have been centrifugally separated (as shown in Fig. 3f). Furthermore, an analysis of histological staining using hematoxylin & eosin (H&E) stain, as well as terminal deoxynucleotide transferase (dUTP) nick end labeling (TUNEL) followed by a blinded analysis by a pathologist was performed. It indicated higher endothelial damage of livers in the 24hCI-pRBC group (as seen in the predominant TUNEL staining of endothelial cells) compared to 24hCI-acellular group where higher damage to hepatocytes (as seen in the predominant TUNEL staining of hepatocytes) was observed. Representative histological micrographs are shown in Fig. 3g. Comparatively, only marginal cell death was observed in the 0hCI-acellular and 0hCI-pRBC groups, indicating sufficient recovery of organs in these groups of livers.

Other functional metrics, blood gases, and chemistries were also measured including pH, bile production, weight gain, Ca^++^, Na^+^, and HCO_3_^-^_,_ and are summarized in Supplementary Fig. S1. We observed significantly lower pH (t = 5 min) in the 24hCI pRBC group of livers (p < 0.05). We also observed significantly higher Na^+^(t = 60, 120, 180 min), but significantly lower Ca^++^ (t = 120, 180 min) in the pRBC groups (p < 0.05). Interestingly, there was no statistical difference in bile production, however, the 24hCI livers perfused with both compositions gained weight, especially in the 24hCI pRBC group of livers (p < 0.05). We also performed experiments to measure lipid peroxidation, which is a marker of oxidative injury to the tissue. We observed no statistical difference between any of the groups (p > 0.05) (Supplementary Fig. S2).

## Discussion

Solid organ transplantation is a lifesaving procedure, yet exposure of grafts to ischemia–reperfusion during transport and implantation may limit its full potential. Machine perfusion (MP) allows minimization of and recovery from IRI when compared to the current clinical standard of static cold storage. Different machine perfusion methods use different temperatures and perfusate compositions. However, the direct impact of various machine perfusion protocols on mitochondrial injury are incompletely understood. To enhance our understanding of mitochondria as a function of cold ischemia and oxygenation strategies during machine perfusion, we leveraged a highly specific, sensitive, and non-destructive method to quantify the redox state of mitochondrial cytochromes with resonance Raman Spectroscopy (RRS). Such technologies may help further optimize conditions of perfusion, provide a better understanding of the underlying mechanisms of IRI, and identify new therapeutic interventions to overcome mitochondrial injury.

We present a platform for non-invasive, real-time assessment of oxygenation of mitochondria during ex vivo liver perfusion using resonance Raman spectroscopy. Through experiments in rat livers, we aimed to provide mechanistic insights into oxygen demand and supply at the electron transport chain during normothermic machine perfusion-based recovery from cold ischemia. For instance, when a minimally ischemic (< 15 min) rat liver is perfused with either an acellular perfusate or packed RBC based perfusate, 3RMR is low, indicating sufficient oxygenation (Fig. 2b). However, when a 24-h cold ischemic (24hCI) liver is perfused, the pRBC perfusate group shows a low 3RMR (< 25, sufficiently oxygenated) while the acellular perfusate group shows a high 3RMR (> 40, insufficiently oxygenated) (Fig. 2c), which is also corroborated by significant hepatocyte death as observed via TUNEL staining. It is possible that there is an accumulation of electrons at the mitochondrial cytochromes during cold ischemia, which can be rapidly transferred to the abundant oxygen molecules in the case of the pRBC-based perfusate. However, this process is comparatively slower in the case of acellular perfusates due to a lower concentration of oxygen. This trend is also supported by the high lactate levels and high oxygen uptake rate in the 24hCI groups immediately upon reperfusion (Fig. 2e,f). Once the organ has stabilized (after t = 30 min), we also see a correlation of 3RMR with oxygen uptake rate and lactate levels in these groups of livers (Supplementary Fig. S3).

While sufficient reoxygenation after ischemia is necessary for recovery, a sudden temperature change and oxygen burst may also exacerbate IRI. Indeed, the injury markers ALT and AST showed higher levels in organs that are perfused with pRBC compared to the organs perfused with an acellular perfusate (Fig. 3b,c). This damage is also confirmed by histology with H&E and TUNEL stains (Fig. 3g), where higher endothelial cell death in livers perfused with packed RBCs is observed as compared to acellular perfusate. Quite interestingly, 3RMR rises transiently at the end of 2.5 h (t = 150 min) of perfusion in the 24hCI pRBC group (p < 0.1 for comparison with 24hCI Acellular group), indicating the possibility of rising injury over time (Fig. 2b). It is possible that a reduced supply of oxygen due to coagulation of pRBCs in the vasculature^30^, RBC damage due to hemolysis, or shrinking of blood vessels due to hyperoxygenation^14,31^ may have reduced local supply of oxygen causing high 3RMR values. It may be possible to reduce some of these effects with the use of acellular perfusates, such as those used in this study. After 3 h of perfusion in the 24hCI Acellular group, the 3RMR value is low (Fig. 2c) with positive indications in other parameters too including stable oxygen consumption (Fig. 2f), and low lactate level (< 2 mmol/L^32,33^) (Fig. 2e). However, acellular perfusates also possess problems such as insufficient oxygenation in the early phase (as seen by the high 3RMR) which may cause further damage, such as observed by hepatocyte cell death in histology (Fig. 3g).

To further improve outcomes, perfusate compositions may be varied in conjunction with gradual re-warming strategies to control the rise in metabolism aspect of recovery. In one such previous study with this technology, 24hCI livers were recovered with a less severe shift in temperature- by perfusion at sub-normothermic temperature (SNMP), which allowed the organ to come to a stable function within 30 min of perfusion^25^, with minimal injury in the 24hCI acellular group. Further, others have also shown the promise of gradual rewarming^34,35^ where organs are reheated at a controlled rate to gradually increase the metabolic activity immediately upon reperfusion, allowing sufficient time for an acellular perfusate to recover the accumulated electrons and minimizing the IRI related damage. Due to its unique ability to capture transient oxygenation patterns that are indicative of whole organ function, 3RMR may indeed provide an opportunity for dynamic optimization of recovery of livers using both controlling rate of rewarming and perfusate composition.

It is important to note the significance of these results in the context of the several limitations of this study. First, the choice of 24-h cold storage duration for rat livers is based on previous experiments by other groups at our center that showed 100% survival post-transplant in this treatment group compared to 48 and 72- hour storage groups^27,36^, although transplantation was not performed in this study. A second important factor that should be considered in the interpretation of these results is the size of the spot (2 mm diameter) and the depth of penetration (estimated to be < 1 mm) of the 441 nm laser used for these measurements. Such a localized measurement prevents us from obtaining a global representation of organ health, as it may fail to capture the damage in other parts of the organ. On the other hand, a local measurement may help us better understand the local biology that would be missed by other global metrics, such as perfusate measurements. Importantly, this problem will be exemplified with large animal models such as swine and human organs, yet there are several ways to address this challenge. For example, biopsies collected from the core of the organ could be used to predict mitochondrial health of deeper tissue or an ultra-thin needle-like probe could be inserted into the organ parenchyma for deeper measurements. Finally, multi-laser technology would enable measurements on a larger surface area that could be used for more global measurements with a single output.

In conclusion, there is an urgent need for mechanistic, non-invasive, and real-time assessment of IRI during machine perfusion due to the low specificity and sensitivity of currently used markers^37^. We successfully developed a novel approach that provides a reporter free highly specific readout of functional oxidation state of mitochondrial cytochromes during machine perfusion in real-time. Better understanding of mitochondrial function using 3RMR may help in better optimization of the strategies for reperfusion, while minimizing injury and maximizing functional recovery before transplant^38^.

## Materials and methods

### Liver procurement and storage

All animals for the experiments were maintained in accordance with National Research Council guidelines and were approved by the Institutional Animal Care and Use Committee (IACUC) at Massachusetts General Hospital (Boston, MA, USA). Female Lewis rats (200 g, Charles River Laboratories) were sedated in an induction chamber using isoflurane set to 3–5%. The excess vapor was filtered through with activated charcoal (Vet Equip Vapor Guard Activated Charcoal, 931401). The animal was then removed from the chamber and placed on a surgical table in a supine position. Depth of anesthesia was deemed adequate when muscular contraction was absent following toe pinch. The abdominal region was shaved, and abdomen is opened with a transverse abdominal incision. The hepatic artery and branches of portal vein were ligated and 200U of sodium heparin (MGH Pharmacy) was injected into the supra-hepatic vena cava. The bile duct was cannulated with a 22G polyethylene tube and a 16G catheter (BD Insyte Authoguard, 381454) was inserted into the portal vein, and the IVC transected. The liver was immediately flushed with 40 mL of heparinized saline solution and an additional 20 mL to remove any residual blood. Perfusion was started within 5 min of harvesting, except for livers exposed to durations of static cold storage events which were further flushed with 25 mL of chilled University of Wisconsin and stored in 50 mL of UW solution at 4 °C. The storage duration of 24 h was used due to the 100% successful transplantation outcomes in this storage duration as observed by other groups in our center, which significantly decline for 48 and 72 h of storage^27,36^.

### Normothermic machine perfusion setup

The perfusion set-up provides oxygenated continuous flow through the portal vein at temperature of 37 °C. The specifics of the machine perfusion protocol used were previously covered elsewhere^39^. Perfusion was flow controlled; the flow gradually increased from 6 ml/min to a maximum perfusion rate of 30 ml/min.

Acellular perfusate was composed of base William’s E medium containing 1.022 g of BSA (Sigma-Aldrich, A7906), 2.4 mg of dexamethasone (Sigma-Aldrich, D2915), 1.02 mL of penicillin streptomycin (Sigma-Aldrich, P4458), 1.02 mL of Glutamax (Thermo Fisher Scientific, 35050061), 0.5U of insulin and 200U of heparin. Perfusate was sterile filtered, and pH stabilized at 7.4 by adding sodium bicarbonate (MGH Pharmacy). For the cellular perfusate group, 80 mL of acellular perfusate was added to 20 mL packed red blood cells (i.e. final hematocrit of 15–20%) to bring total volume up to 100 mL. Prior to perfusion of the liver, the perfusate was cycled for about 15 min in the perfusion system to oxygenate and warm it to 37 °C.

### Red blood cell collection and preparation of cellular perfusate

The donor rats were anesthetized by inhalation of isoflurane (3–5%) in an induction chamber and depth of anesthesia was confirmed by lack of response following toe pinch. The animal was then placed on heating pad in a supine position, and the area around the lower rib was shaved. A small midline incision was made, and the underlying tissue was separated. The heart was located and a 23G needle attached to a 10 mL syringe containing sodium heparin was inserted into the left ventricle, and the slowly retracted until no blood was available. A total of 6-8 mL of blood was collected and the rat was euthanized by exsanguination.

Whole blood collected in sodium heparin was spun down at 2200*g* for 10 min at 20 °C. The plasma and buffy coat layers are aspirated, and red blood cells are resuspended in perfusate and spun down two more times for the wash process. The washed RBCs are transferred into sterile tubes and stored at 4 °C for up to 5 days.

### Resonance Raman spectroscopy setup

The portable RRS System (Pendar Technologies) was developed as a compact 441 nm laser device housing a power laser source of 8.9mw and compact probe with a flexible fiber optic bundle. The probe head is an 8 mm × 12 mm × 60 mm which delivers single mode laser light about 2 mm diameter on the tissue. The light is passed through the collection optics, and the filtered RR photons are collected at the temperature-controlled two-dimensional charge coupled device (CCD) detector array. Measurements are recorded for total time of 180 s with isolated signals from mitochondrial in the 700–1700 cm^−1^ spectral range. The readout from the CCD is recorded and analyzed using custom LabView software. We used the following libraries for this analysis: mito-reduced and oxidized, cytochrome c reduced and oxidized, residual 1592 (i.e., rat liver specific background) library, and for pRBC groups- hemoglobin reduced and oxidized. Measurements are made by focusing the laser on a spot on the left lateral lobe in the region that is in proximity to the portal vein. This region of the liver was found in a previous study^25^ to be an optimum predictor of organ function, and hence all the measurements for all groups are made at this location.

### Experimental design

Our experimental groups that consist of 0hCI and 24hCI livers were perfused with perfusates carrying a low and high supply (acellular and pRBC perfusate) of oxygen as summarized in Fig. 2a. Thus, a total of four groups (0hCI Acellular, 0hCI pRBC, 24hCI Acellular, 24hCI pRBC), with each group consisting of 3–4 replicates. Within each group, we obtained the 3RMR value every 30 min starting roughly 5 min after the beginning of machine perfusion. We also performed an analysis of the blood gases and chemistry of the perfusate at every hour of perfusion to validate the perfusion technique. At the end of the perfusion, wedge biopsies were flash frozen for energetic analysis including ATP, ADP, AMP ratios, and NAD:NADH ratio that show mitochondrial electron transport chain activity both downstream (i.e., at complex V) and upstream (i.e., at complex I) of 3RMR. Finally, we also compared injury to the graft using markers including flow resistance during perfusion, hemolysis, potassium level, ALT/AST levels every 60 min during perfusion, and histological markers of injury at the end of perfusion.

### Perfusion data acquisition and processing

Blood gas analysis by perfusate sampling every thirty minutes using the RAPIDPOINT 500 Blood gas analyzer (Siemens Healthineers, Munich, Germany) for measurements of blood gases and chemistries including pH, pO2, electrolytes (Na^+^, K^+^, Ca^++^, CI^−^), and metabolites (lactate). AST and ALT were measured hourly from the venous outflow using the Piccolo Xpress Chemistry Analyzer (Abbott, Illinois, USA). For hematological analysis, perfusate samples collected were centrifuged at 4000*g* for 10 min. Supernatant were collected and stored at − 20 °C which were later recovered for free hemoglobin assessment using the NanoDrop-One Microvolume UV–Vis Spectrophotometer (ThermoFisher Scientific, Waltham, MA, USA) at 414 nm. Hematocrit was checked prior to perfusion using Sysmex XP-300™ Automated Hematology Analyzer (Sysmex, Kobe, Japan).

The liver was weighed immediately after harvesting and after machine perfusion. The pressures and flow rates were recorded every 30 min during the 3 h perfusion run. Liver tissues were flash frozen in liquid nitrogen immediately after perfusion and stored at − 80 °C. The concentrations of NAD, NADH, NADP and NADPH in liver tissue were analyzed by mass spectrometry core of Shriners’ Children’s Boston (Boston, MA, USA). MDA concentration was also measured in this tissue by using the lipid peroxidation kit (MAK085) from Millipore Sigma. MDA concentration per sample was normalized with the total protein concentration in the same measured using Pierce BCA Protein Assay Kit from ThermoFisher Scientific (Catalog # 23227). Liver biopsies were also fixed in 10% formaldehyde for a maximum of 96 h before transferring into 70% ethanol. Fixed tissues were processed for TUNEL and H&E staining, which was performed by the Massachusetts General Hospital Histology Molecular Pathology Core (Charlestown, MA, USA).

### Statistical analysis

All statistical analyses were performed in Prism 9 (GraphPad Software Inc., La Jolla, CA). One-way and two-way ANOVA based Sidak multiple comparison test in GraphPad were used to derive statistical conclusions. Since only an n of 3–4 was used per group, we plotted and used median ± interquartile range for all measurements across all groups as represented in all figures and text of the manuscript. Correlation were performed using Spearman’s correlation test with one tailed analysis for these variables.

### Supplementary Information

Supplementary Figures.

## Data Availability

The authors affirm that the primary data supporting the conclusions of this research are represented within the article. Any other information not contained within the article will be made available by the corresponding author upon reasonable request.
