# Supplementary material for: Real-time monitoring of mitochondrial oxygenation during machine perfusion using resonance Raman spectroscopy predicts organ function
Source: Sci Rep. 2024 Mar 27;14:7328. doi: 10.1038/s41598-024-57773-w (PMC10973340; doi:10.1038/s41598-024-57773-w)
Supplement: Supplementary file 1 — Supplementary Figures. [file 41598_2024_57773_MOESM1_ESM.pdf]

SUPPLEMENTARY FIGURES

Supplementary Figure 1: Perfusion parameters.

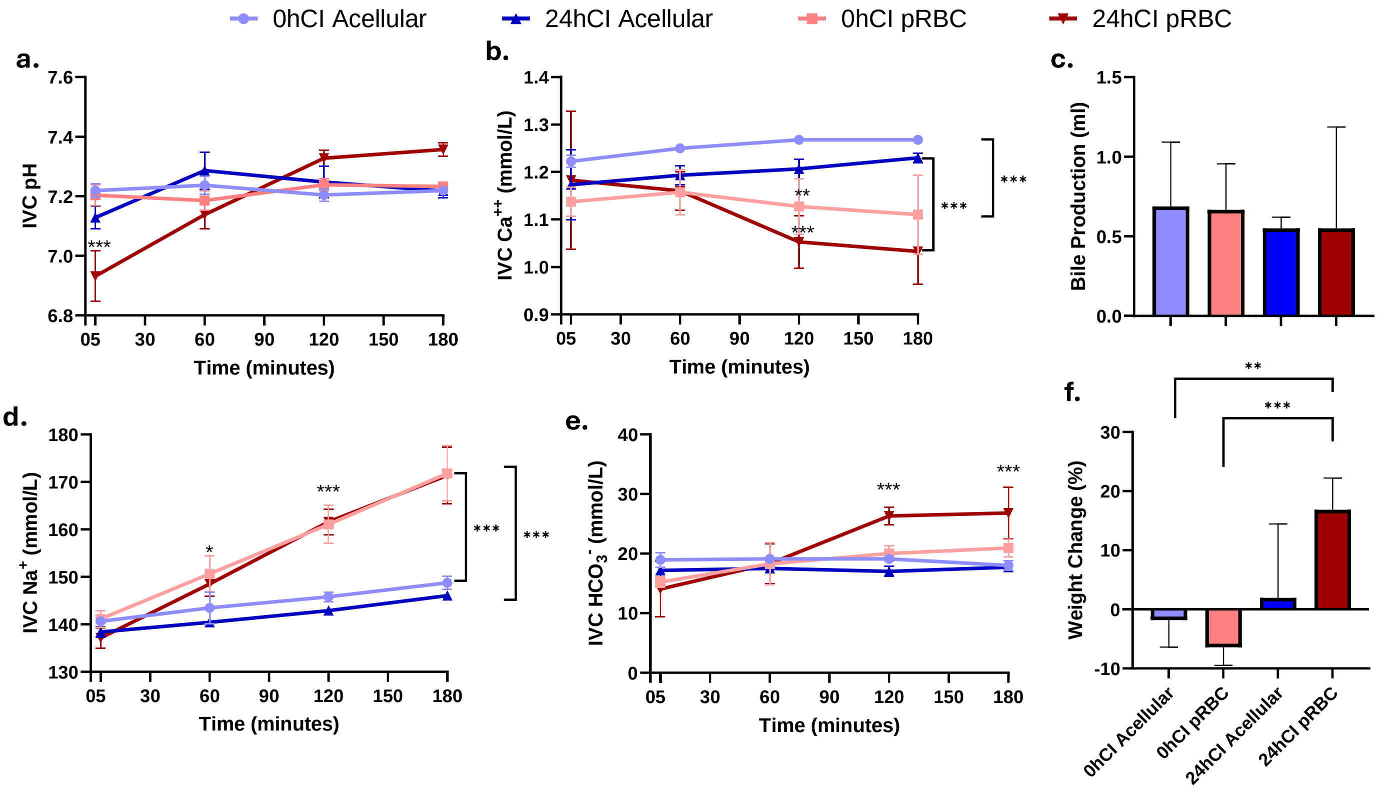

Markers of perfusate biochemistry, bile production and weight change. Perfusate biochemistry was monitored throughout the duration of perfusion as explained in methods section for IVC outflow pH, IVC Ca^{++}, IVC Na^{+}, and IVC HCO_3^-. We also quantified bile production and weight change of the whole liver. n=3-4 for each group. Statistically significant differences are shown as follows- * $p<0.05$, ** $p<0.01$, *** $p<0.005$.

Supplementary Figure 2: Lipid peroxidation using malondialdehyde (MDA) levels.

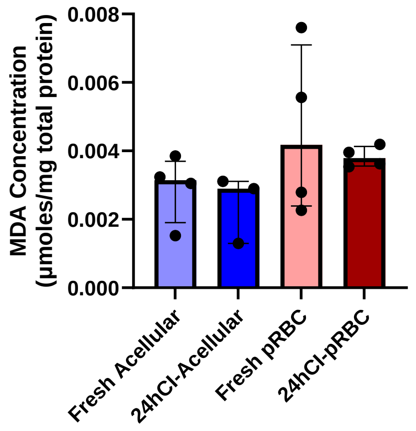

Measurement of lipid peroxidation as a marker of oxidative stress. A comparison of malondialdehyde (MDA) concentration, which is a measure of lipid peroxidation in biological material, was performed to assess damage due to oxidative stress. No statistically significant differences were observed between any of the groups ($p>0.05$, n=3-4).

Supplementary Figure 3: Correlation of 3RMR with perfusion metrics.

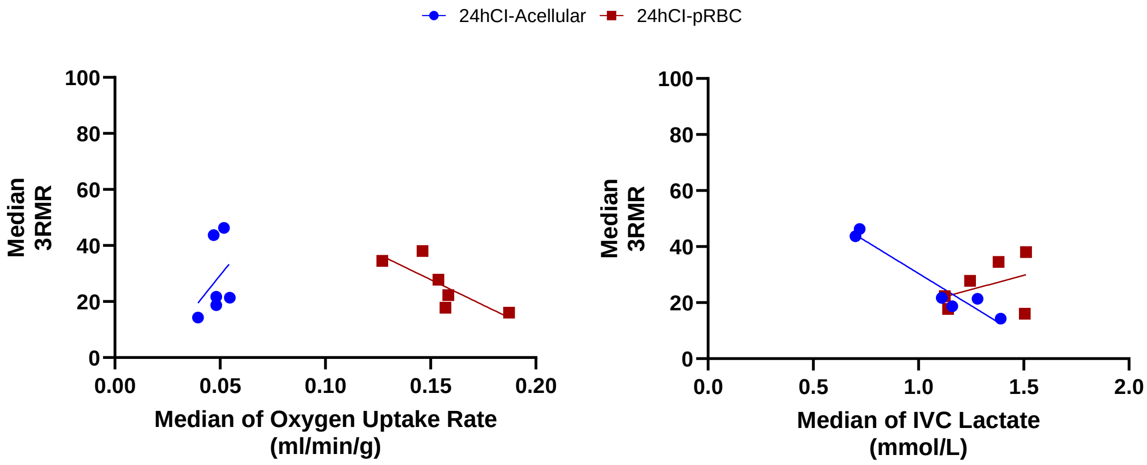

Condition	Spearman r	p-value
24hCI-Acellular	0.43	0.21
24hCI-pRBC	-0.89	0.02

Condition	Spearman r	p-value
24hCI-Acellular	-0.89	0.02
24hCI-pRBC	0.37	0.25

Correlation assessment between 3RMR with Oxygen Uptake Rate and IVC lactate for cold stored livers. We performed linear regression with spearman's rank correlation analysis to study the relationship between 3RMR with both oxygen uptake rate, and IVC lactate. Median values at each time starting t=30 minutes from 3-4 replicates for each group were used for this analysis in GraphPad. Graphs above show the results of this analysis with individual plots for each condition and a table that summarizes the results of the correlations observed.
